# Improved Ant Algorithms for Software Testing Cases Generation

**DOI:** 10.1155/2014/392309

**Published:** 2014-05-05

**Authors:** Shunkun Yang, Tianlong Man, Jiaqi Xu

**Affiliations:** School of Reliability and Systems Engineering, Beihang University, Beijing 100191, China

## Abstract

Existing ant colony optimization (ACO) for software testing cases generation is a very popular domain in software testing engineering. However, the traditional ACO has flaws, as early search pheromone is relatively scarce, search efficiency is low, search model is too simple, positive feedback mechanism is easy to porduce the phenomenon of stagnation and precocity. This paper introduces improved ACO for software testing cases generation: improved local pheromone update strategy for ant colony optimization, improved pheromone volatilization coefficient for ant colony optimization (IPVACO), and improved the global path pheromone update strategy for ant colony optimization (IGPACO). At last, we put forward a comprehensive improved ant colony optimization (ACIACO), which is based on all the above three methods. The proposed technique will be compared with random algorithm (RND) and genetic algorithm (GA) in terms of both efficiency and coverage. The results indicate that the improved method can effectively improve the search efficiency, restrain precocity, promote case coverage, and reduce the number of iterations.

## 1. Introduction

Software testing is the implementation of the system and components and the behavior of observing and recording results under given conditions [1]. Test cases (TC) set plays an important role in the process of software testing and determines the quality of software directly. TC in software testing is a data set, such as input data, execution paths, execution conditions, and testing requirements [2]. For a long time, the TC generation mainly relied on manpower, which means that software testers need to have rich experience in software testing. At the same time, it also leads to the fact that the actual engineering of TC generation work often has great blindness and some problems emerge, such as the huge TC number, high manpower cost, hardly ascend test case coverage, and so forth. Therefore, how to automatically generate TC scientifically and efficiently becomes the research focus of software testing engineer. Over the years, many researchers in automatic TC generation field have carried out extensive and in-depth research and have got a lot of research results. In 1992, ant colony optimization is proposed by Dorigo et al. [3] in his Ph.D. degree thesis. The inspiration comes from the process of ant colony searching for food. Ant colony optimization is a kind of simulated evolutionary algorithm; preliminary studies show that the algorithm has a good optimization performance. And it has many advantages, such as easy combination with other algorithms and strong robustness and adaptive self-organizing ability. In 2003, McMinn and Holcombe [4] introduced heuristic ant colony optimization into the software test case generation technology for the first time. As the ant colony algorithm is being used widely gradually in the test case generation technology, many tools for software testing based on ACO have been developed. Among them, PPTACO for optimal path generation [5], STTACO for test sequence generation [6], EPPTACO for path generation [7], and PCTDACO for test data generation [8] are the representative tools. Recently, Sharma et al. [9] developed another tool, ESCov, which is based on ACO generation state transition test sequence and achieves maximum coverage and minimum redundancy. After that, Suri and Singhal [10] proposed ACO_TCSP tool which is based on ACO algorithm to select TC and greatly reduces the number of cases to 62.5%. Not long after, Suri and Singhal [11] considered execution time constraints and accuracy, in the eight selected programs, and most of the TC numbers decreased by more than 80%. And some scholars apply ACO to many aspects of test data generation technology. Singh et al. [12] applied ACO on test case prioritization. Before long, Bauersfeld et al. [13] applied ACO to input queue of graphical user interface (GUI) generated automatically and applied dynamic feedback between the system under test (SUT) and the search process. Recently, Srivastva et al. [14] put forward a kind of nonrepeated state transformation method based on ACO algorithm; this method reduces the redundant state conversion and effectively covers the status of the transformation uncovered. And Singh et al. [15] compared a white box testing technology based on ACO algorithm with the existing black-box testing technique and came to the conclusion that path coverage based on ACO algorithm is higher. Now, ant colony optimization has a wide application in test case generation technology. But because of the insufficiency of the algorithm theory itself, it produces many problems, as early search pheromone is relatively scarce, search efficiency is low, search model is too simple, positive feedback mechanism is easy to produce stagnation and precocity phenomenon. In order to overcome the shortage of the ant colony optimization, in this paper, we put forward ILPACO, IPVACO, IGPACO, and ACIACO and build ant colony research path model to improve the search efficiency, restrain precocity, promote case coverage, and reduce the number of iterations.

## 2. The Description of the Improved Ant Colony Optimization

Ant colony optimization has a good optimization effect on path optimization, especially traveling salesman problem (TSP) [16–18]. In this paper, we construct ant colony path model to realize the software test case generation. Using the path model presented in this paper, because of a lack of closed loop feedback, the simple ACO (SACO) will tend to move randomly. We improve ant colony optimization to realize generation of higher coverage TC and prove that the improved algorithm is effective for test case generation. In Section 2, we will describe the four improved ACO algorithms: ILPACO, IPVACO, IGPACO, and ACIACO.

### 2.1. Improve Local Pheromone Update Strategy for Ant Colony Optimization (ILPACO)

SACO is an approach, which uses a small constant to update local pheromone. Let *k* be an integer, 1 ≤ *k* ≤ 2*N* − 1. We denote by *v*_*k*_ path node of ant colony and by *τ*(*v*_*k*_, *v*_*k*+1_) pheromones from node *v*_*k*_ to node *v*_*k*+1_. We define local volatilization coefficient, *α*, and define the small constant to update local pheromone, *τ*_0_. Based on these, the update process of *τ*(*v*_*k*_, *v*_*k*+1_) is defined as follows:

(1) $$\begin{array}{c} \tau \left(v_{k},v_{k+1}\right)⟵\left(1-\alpha \right)\tau \left(v_{k},v_{k+1}\right)+\alpha \tau_{0}. \end{array}$$

In Section 2.1, we present an improved local pheromone update strategy for ant colony optimization (ILPACO). Using the method of program instrumentation, we get the coverage to update local pheromone instead of the small constant *τ*_0_ [19]. Because the value of the coverage will be smaller than one, it is necessary for us to select a proportionality coefficient *k*′ to multiply by the coverage to fit with a wider range of applications. So local pheromone update variable *τ*′ is defined as *τ*′ = *k*′ · coverage. Based on these, the update process of *τ*(*v*_*k*_, *v*_*k*+1_) is defined as follows:

(2) $$\begin{array}{c} \tau \left(v_{k},v_{k+1}\right)⟵\left(1-\alpha \right)\tau \left(v_{k},v_{k+1}\right)+\alpha \tau^{\prime }. \end{array}$$

The coverage presented can be any kind of coverage. In this paper, we select three commonly used kinds of coverage; statement coverage (SC), branch coverage (BC), and modified condition/decision coverage (MC/DC) [20].

### 2.2. Improved Pheromone Volatilization Coefficient for Ant Colony Optimization (IPVACO)

In this section, we describe IPVACO algorithm. For smaller volatilization coefficient *α*, the pheromone volatilization is slower; the optimal path will be restrained. In contrast, for the larger volatile coefficient, but not too large, pheromone evaporates quickly and the effect of the optimal path is strengthened. Larger *α* makes the previous experience to be ignored easily and tend to search more by recent experience. In the SACO algorithm, *α* is fixed. In this paper, we put forward the adaptive *α*, beginning with a small value tendency to search, and the late tendency to develop with larger values. At the same time, this improved method still uses coverage to update local pheromone.

Let *α*(0) be initial volatile coefficient and let *λ* be a constant value. We denote by *t* iteration and *α*(*t*) volatile coefficient of the number *t* iteration. Consider the following:

(3) $$\begin{array}{c} \alpha \left(t+1\right)=\lambda \alpha \left(t\right). \end{array}$$

**Figure 1:**
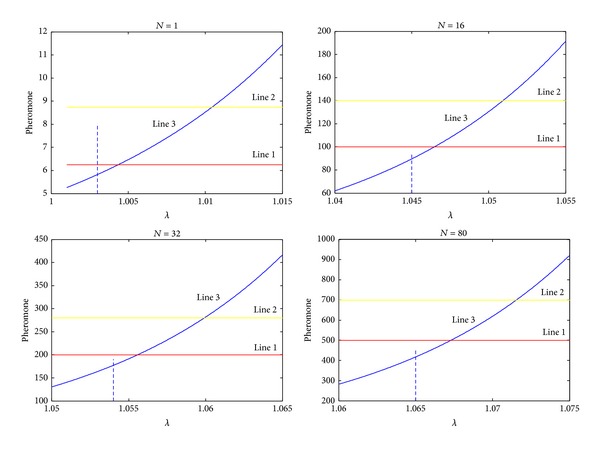
Pheromone volatilization and accumulated certain *λ*.

We need to determine right range of *λ*. Based on the formula (3), it is easy to deduce formula *α*(*t*) = *λ* ^*t*−1^ *α*(1), and total volatile pheromone of one route is *α*(1)·(1 − *λ* ^*t*^)/(1 − *λ*). The value of desired pheromone of one route is *t* · *e* · *N*/4 and *e* is the value of desired pheromone in one generation. Because most of ants' coverage ranges from 0.25 to 0.35, we can select a value range from 0.25 to 0.35. And *N* is the number of ants that update pheromone. In this paper, we select 1, 16, 32, and 80 as *N*. The initial pheromone value of each route is taken as 0.1. Let *α*(1) = 0.05. In Figure 1, line 1 shows the total desired amount of pheromone in one route when *e* is equal to 0.25, line 2 shows the total desired amount of pheromone in one route when *e* is equal to 0.35, and line 3, the exponential curve, depicts the amount of pheromone volatilization with different value of *λ*. Because the pheromone volatilization should be less than the total amount of the path pheromone, according to Figure 1, we get Table 1.

**Figure 2:**
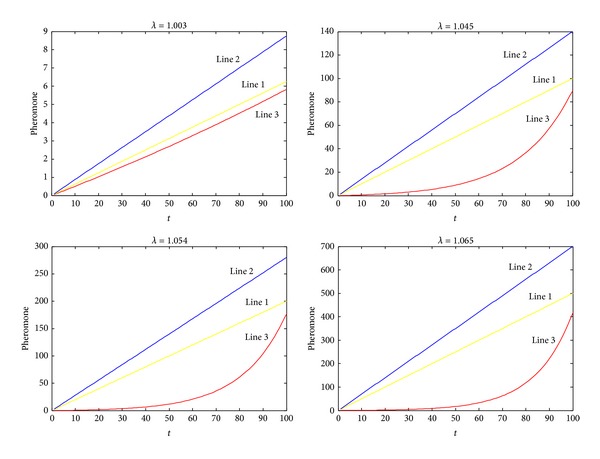
Pheromone lines with different *λ*.

Because we select the value of *λ* in a range, we are not sure whether the value satisfies the condition that the pheromone volatilization should be less than the total amount of the path pheromone. So it is necessary for us to verify if the value is appropriate. we draw Figure 2 to verify the value. It is the same that line 1 shows the total desired amount of pheromone in one route when *e* is equal to 0.25, line 2 shows the total desired amount of pheromone in one route when *e* is equal to 0.35, and line 3, the exponential curve, depicts the amount of pheromone volatilization with increasing generation number.

In Figure 2, we see that line 1 is higher than line 3, which means that amount of the path pheromone is always more than the pheromone volatilization. So we verify that the values in Table 1 are appropriate.

### 2.3. Improve the Global Path Pheromone Update Strategy for Ant Colony Optimization (IGPACO)

**Figure 3:**
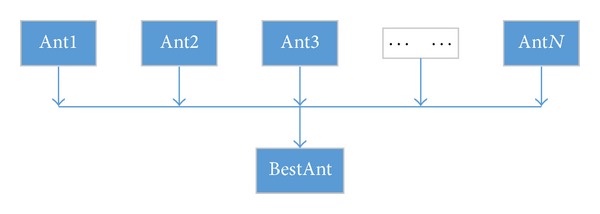
Best ant selection.

SACO algorithm uses all the ants to update pheromone. In Section 2.3, we describe IGPACO algorithm, which is based on ILPACO algorithm. We use the coverage to update local pheromone, at the same time, and only the best ant can update the amount of pheromone in each iteration, rather than all the ants [21]. Performance of ant colony optimization, as a swarm intelligence algorithm, depends on the individual ants and overall synergy effect [22]. According to pheromone concentration distribution, ants choose routes. That makes the convergence speed slow. Besides, there are certain possibilities that ant colony fall into local optimal solution prematurely. We put forward the approach that the optimal ant update pheromone can reduce the branch paths effectively, whose coverage is low, make ants avoid unnecessary paths, and improve the convergence speed.  Figure 3 shows the process of selecting the best ant from *N* ants. Consider the following:

(4) $$\begin{array}{c} \tau \left(v_{k},v_{k+1}\right)⟵\{\begin{array}{cc} \left(1-\alpha \right)\tau \left(v_{k},v_{k+1}\right)+\alpha \tau^{\prime \prime } & \\ \;\;\;\text{if}\;\text{AntI}\;\text{is}\;\text{Best}\;\text{Ant}\left(1\leq I\leq N\right) & \\ \left(1-\alpha \right)\tau \left(v_{k},v_{k+1}\right)\;\text{else}. & \end{array} \end{array}$$

### 2.4. Comprehensive Improved Ant Colony Optimization (ACIACO)

**Figure 4:**
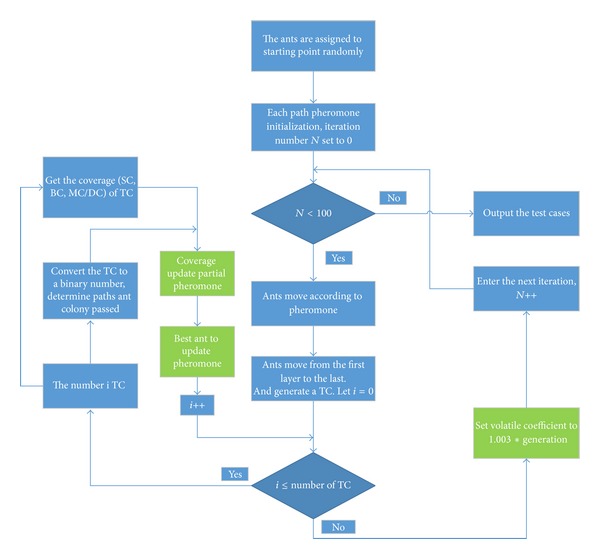
The flowchart of ACIACO.

Comprehensive approach combines together methods represented above comprehensively to improve simple ant colony algorithm. The methods represented conclude ILPACO, IPVACO, and IGPACO. ILPACO, as it shows in (2), can strengthen the ant colony path with high coverage. And the higher coverage the path has, the greater the path's pheromone is, which makes routes have a good gradation with each other [23]. IPVACO, as it shows in (3), improves volatile coefficient increases gradually with the increase of number. Beginning with a small value tend to search and the late tend to develop with larger values. IGPACO selects the best ant update path pheromone in each iteration to search for a more optimal path and improve the convergence speed.  Figure 4 shows the flowchart of ACIACO and the green parts are the parts improved by the optimization algorithms.

## 3. The Construction of the TC-Oriented Ant Colony Path Model

In this work, we establish an effective ant colony optimization path. We utilize this path model to achieve effective iteration. And we explain the transformation relationship between ant colony paths and test cases. The establishment of transformation relationship makes effective use of ant colony algorithm for iterative optimization of software test cases.

### 3.1. The Transformation Relationship between Ant Colony Paths and Test Cases

**Figure 5:**
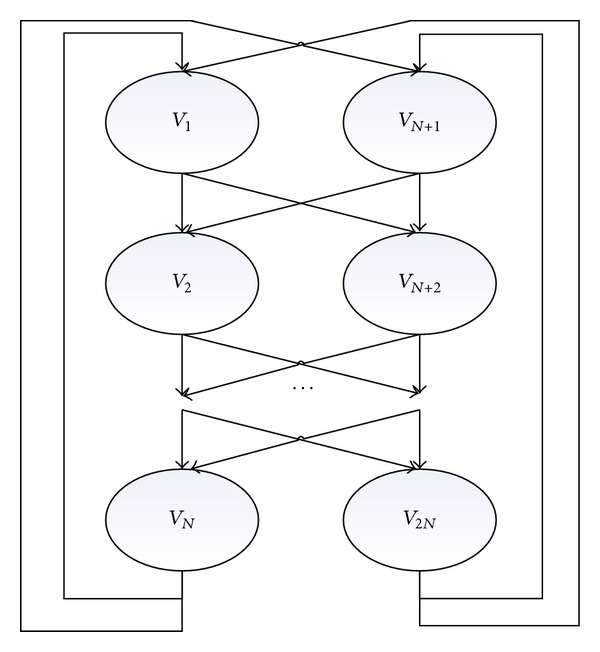
Ant colony path model.

As shown in Figure 5, we define ant path directed graph by *G* = (*V*, *E*) [24]. We describe node set with *V* = {*v*_1_, *v*_2_, *v*_3_,…, *v*_2*N*_} and directed edge set with *E* = {*e*_1_, *e*_2_, *e*_3_,…, *e*_4*N*_}. We build the path model with *e*_1_ = (*v*_1_, *v*_2_), *e*_2_ = (*v*_1_, *v*_*N*+2_), *e*_3_ = (*v*_*N*+1_, *v*_2_), and *e*_4_ = (*v*_*N*+1_, *v*_*N*+2_),…, *e*_4*N*_ = (*v*_2*N*_, *v*_*N*+1_). Ant colony optimization path is an *N* layer structure; each layer has two nodes, a total of 2*N* nodes. There are four paths between every adjacent two layers, a total of 4*N* paths. Set value in 1 ~ *N* nodes to 0 and value in *N* + 1 ~ 2*N* nodes to 1. Ants' every movement can only choose one node in each layer. According to the pheromone, ants follow the arrow direction to move to the next layer and complete the movement of the *N* layer structure. Then, ants move back to the first layer of the path and begin movement of the next generation until the end of the iterations. According to the route of the last generation of ants, which equals to the number the *N* bit binary number. Through the antispoofing, we get the test case.

**Figure 6:**
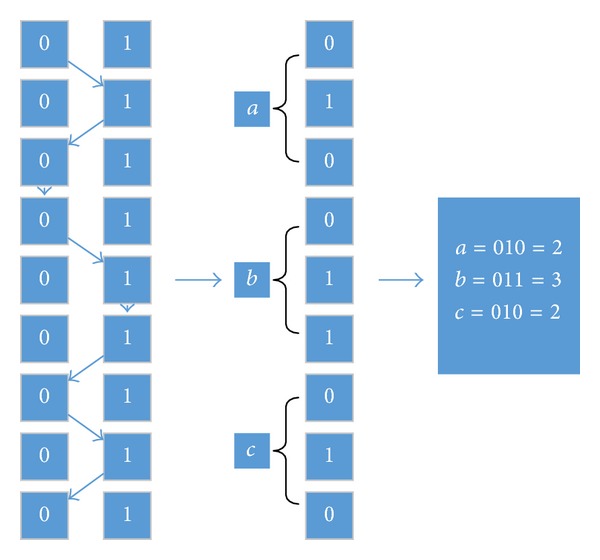
The transformation relationship between ant colony paths and test cases.

As shown in Figure 6, we give a simple example to explain the relationship between the path and test case. The undertest program is the triangle determination program; the data of test case are three lines of triangle, *a*, *b*, and *c*. We cut the *N* bit binary number into three *N*/3 parts: the first part corresponding with *a*, the second part corresponding with *b*, and the third part corresponding with *c*. Generally, we can change the value of *N* and the value of nodes to satisfy different-type and different-interval test cases. The last generation of ants is the eventually generated test cases and the ant number is equal to the number of test cases at last generation.

### 3.2. Ants Movement Rules

In Section 3.2, we explain the ant movement rules. Firstly, we take a small constant to initialize ant colony path pheromone. According to (1), ants in the current node *v*_*j*_′ = *v*_*k*_(1 ≤ *k* ≤ 2*N*) can only move to one of two nodes in the next layer, *v*_*j*+1_′. We define *τ*(*v*_*k*_, *v*_*k*+1_) pheromone of the directed line *e*(*v*_*k*_, *v*_*k*+1_); *q* is a random number from the range [0,1].

If 1 ≤ *k* ≤ *N* − 1,

(5) $$\begin{array}{c} v_{j+1}^{\prime }=\{\begin{array}{cc} v_{k+1}, & q\leq \frac{\tau \left(v_{k},v_{k+1}\right)}{\left(\tau \left(v_{k},v_{k+1}\right)+\tau \left(v_{k},v_{k+1+N}\right)\right)}\\ v_{k+1+N}, & \text{else}. \end{array} \end{array}$$

If 1 + *N* ≤ *k* ≤ 2*N* − 1,

(6) $$\begin{array}{c} v_{j+1}^{\prime }=\{\begin{array}{cc} v_{k+1}, & q\leq \frac{\tau \left(v_{k},v_{k+1}\right)}{\left(\tau \left(v_{k},v_{k+1}\right)+\tau \left(v_{k},v_{k+1+N}\right)\right)}\\ v_{k+1+N}, & \text{else}. \end{array} \end{array}$$

If *k* = *N* or *k* = 2*N*,

(7) $$\begin{array}{c} v_{j+1}^{\prime }=\{\begin{array}{cc} v_{1}, & q\leq \frac{\tau \left(v_{k},v_{1}\right)}{\left(\tau \left(v_{k},v_{1}\right)+\tau \left(v_{k},v_{N+1}\right)\right)}\\ v_{1+N}, & \text{else}. \end{array} \end{array}$$

## 4. Experimental Analysis

In this section, we present the result of the four proposed approaches. C++ language is used for experiment programming. Classic triangle classification (CTCP) and the collision avoidance system (TCAS) are selected as two undertest programs. Because CTCP and TCAS have characteristics of many branches and determines, they are suited as undertest programs for software test case generation. In Section 4.1, we introduce the experiment process of CTCP. And, in Section 4.2, we introduce the experiment process of TCAS. In two experiments, we compare data by seven approaches, including RND, GA, SACO, ILPACO, IPVACO, IGPACO, and ACIACO. We use the statement coverage, branch coverage, and modified condition/decision coverage (MC/DC), three kinds of coverage, as quality standard of test cases. In order to avoid the contingency, we select average coverage and average minimum generation of all test inputs in 100 runs as the experimental data [25]. In Section 4.3, we conclude and analyze the efficiency of all improved approaches.

### 4.1. Classic Triangle Classification Experiment

The lines of classic triangle classification program are 119. Cyclomatic complexity is 19. In GA, we determine the GA parameters (maximum number of generations (MaxGens), population size (PopSize), crossover probability (XP), mutation probability (MP), and select probability (SP)). We set the parameters of GA as MaxGens = 100, PopSize = 50, XP = 0.1, MP = 0.1, and SP = 0.5. Let volatile coefficient *α* = 0.05 and let proportionality coefficient *k* = 1.0. In volatile coefficient schedule, let *λ* = 1.003 for IGPACO and ACIACO, the corresponding value in Table 1 for others, and *α*(1) = 0.05. The total number of iterations is 100.

Table 2 shows three kinds of coverage of the seven approaches: RND, GA, SACO, ILPACO, IPVACO, IGPACO, and ACIACO. The number of ants is set to the value 16. RND and GA both generate 16 test cases. Because of SACO's lack of coverage feedback, it is similar to RND. And we find that GA is similar to IGPACO. The statement coverage of ACIACO achieves 100%.

Table 3 shows changes of coverage by choosing different numbers of ants on ACIACO. We select the number of ants 8, 16, 24, 32, 40, 48, 56, 64, and 72, respectively. We also use statement coverage, branch coverage, and MC/DC as quality standard of test case. As shown in Table 3, the number of ants is 16 and the statement coverage achieves 100%. When the number of ants is increased to 32, statement coverage and branch coverage reach 100%. Because MC/DC interaction intensity is high, when the number of ants arrived at 56, the coverage only stops at about 89%. Unless a large number of ants increase, it is difficult to improve MC/DC coverage. How to improve the MC/DC, high interaction intensity coverage, we need new improved ant colony algorithm to achieve it.

Table 4 shows, when branch coverage achieves 100%, the average minimum generation on seven approaches. As shown in Table 3, the number of ants is 32 and ACIACO achieves 100%. So we select the ant number 32. We can see that RND and SACO do not achieve 100%, even more than 10000 generations. GA and IGPACO are similar. IGPACO can reduce number of iterations very well; the average minimum generation is reduced from 161.34 to 47.89. The minimum average iteration number of ACIACO is 24.12. ACIACO reduces the iteration number greatly.

### 4.2. The Collision Avoidance System

The lines of the collision avoidance system are 213 and cyclomatic complexity is 19. In GA, we set the parameters of GA as MaxGens = 100, PopSize = 50, XP = 0.1, MP = 0.1, and SP = 0.5. Program generates twelve aircraft flight parameters. The number of ant colony network nodes is set to 118, the number of the route model on one side is 59, and, thereinto, the parameter Cur_Vertical_Sep is 1st~11th node; High_Confidence is boor type, so it is 12th node; Two_of_Three_Reports_Valid is boor type; it is 13th node; Own_Tracked_Alt is 14th~24th node; Own_Tracked_Alt_Rate is 25th~30th node; Other_Tracked_Alt is 31st~35th; Alt_Layer_Value is 36th~37th node; Up_Separation is 38th~45th node; Down_Separation is 46th~53rd node; Other_RAC is 54th~55th node; Other_Capability is 56th~57th node; Climb_Inhibit is 58th. Let volatile coefficient *α* = 0.05; proportionality coefficient *k* = 1.0. In volatile coefficient schedule, let *λ* = 1.003 for IGPACO and ACIACO, the corresponding value in Table 1 for others, and *α*(1) = 0.05. The total number of iterations is 100.

Table 5 shows three kinds of coverage of the seven approaches: RND, GA, SACO, ILPACO, IPVACO, IGPACO, and ACIACO. The number of ants is set to 16. At the same time, RND and GA both generate 16 test cases. In the same way, RND is similar to SACO. In ACIACO, statement coverage achieves 92.90%, branch coverage achieves 75.11%, and MC/DC achieves 33.64%. Through analyzing data in Table 5, GA is similar to IPVACO and IGPACO. And three kinds of coverage of ACIACO are highest.

Table 6 shows changes of coverage by choosing different numbers of ants on ACIAC. We select the number of ants 8, 16, 24, 32, 40, 48, 56, 64, 72, 80, and 88, respectively. We also use statement coverage, branch coverage, and MC/DC as quality standard of test case. When the number of ants is 64, the statement coverage achieves 100%. When the number of ants increases to 16, the statement coverage and branch coverage achieve 100%. We find that when the numbers of ants are 32, 40, and 48, the three kinds of coverage increase slowly, even decrease. That is a normal phenomenon. Some parts hardly be covered need test cases those appear with low probability to cover. There is a situation that 40 ants have a higher coverage than 48 ants do. The reason is that 48 ants do not conclude those test cases hardly be covered, but 40 ants conclude part of them. And when the number of ants increases to 48, some test cases generated with low possibility have been generated continually and coverage increases again. All the same, because MC/DC interaction intensity is high, when ants arrived at 80, the coverage only stops at about 76%.

As is shown in Table 7, we select the number of ants as 80. And, in GA, the number of test cases is 80. We conclude the average minimum generation number on seven approaches, whose branch coverage achieves 100%. We get some similar conclusions. RND and SACO do not achieve 100%, even more than 10000 generations. GA is similar to IGPACO. IGPACO can reduce number of iterations very well; the average number of iterations is reduced from 238.34 to 96.39. IGPACO is better than IPVACO. The average minimum iteration number of ACIACO is 28.24. ACO greatly reduces number of iterations.

### 4.3. Discussion and Analysis

From the above experimental data, we can conclude the performance of seven different algorithms. RND and SACO are random processes and the coverage is lowest. GA is effective to promote coverage of test cases and decreases number of iterations. And the performance of GA is similar to that of IGPACO. ILPACO uses coverage as ant colony pheromone feedback to strengthen the advantage path and coverage of test cases is promoted. Based on ILPACO, IPVACO uses adaptive volatile coefficient and makes volatile speed slow first and then faster. The coverage of test cases generated on IPVACO is better than ILPACO. IGPACO uses best ant to update pheromone. IGPACO has a good astringency. And the minimum number of iterations where branch coverage achieves 100% is much smaller than IPVACO and IGPACO. ACIACO, which is based on all the above three methods, can effectively improve the search efficiency, restrain precocity, achieve the highest coverage, and minimize the number of iterations. The proposed technique will be compared with RND and GA in terms of both efficiency and coverage. The results indicate that the improved method can improve the search efficiency, restrain precocity, promote case coverage, and reduce the number of iterations effectively.

## 5. Conclusions

In this paper, we have improved ACO, establish ant colony search path, and put forward improved approaches: ILPACO, IPVACO, IGPACO, and ACIACO. The proposed technique is compared with RND and GA in terms of both efficiency and coverage. Through the comparison and analysis of CTCP and TCAS, the improved method can effectively improve the search efficiency, restrain precocity, promote test cases coverage, and reduce the number of iterations.

The future work will consider following fields. (1) Construct high efficiency ant colony search path. Because ant colony path model is simple, the algorithm astringency is reduced. Establishing efficient ant colony search path can significantly improve test case coverage and reduce the number of iterations greatly. (2) Improve ant colony algorithm further sophisticatedly. For MC/DC such correlation extremely high coverage, we need to think of a more sophisticated algorithm; algorithm should take into account correlation problem of the structures and variables in under test program. (3) Based on ACO, put forward the comprehensive algorithm with other intelligent optimization algorithms. Because of some disadvantages in ant colony optimization, we can use genetic algorithm, particle swarm optimization, artificial bee colony, or another heuristic algorithm to combine with ant colony optimization effectively, mutually complementing each other. The comprehensive algorithm will improve the quality of the software test case generated effectively.

## Figures and Tables

**Table 1:** The value of *λ* corresponding to different *N*.

*N* = 1	*λ* = 1.003
*N* = 16	*λ* = 1.045
*N* = 32	*λ* = 1.054
*N* = 80	*λ* = 1.065

**Table 2:** Coverage of CTCP by seven approaches.

	Statement coverage	Branch coverage	MC/DC
RND	62.25%	47.37%	11.90%
GA	92.45%	77.84%	22.76%
SACO	61.78%	49.58%	14.03%
ILPACO	67.98%	54.84%	22.90%
IPVACO	88.28%	75.69%	22.88%
IGPACO	90.34%	75.49%	23.45%
ACIACO	100.00%	88.83%	46.34%

**Table 3:** The coverage of different ant numbers on ACIACO.

ACIACO	Statement coverage	Branch coverage	MC/DC
8 ants	92.23%	80.23%	33.98%
16 ants	99.65%	88.34%	44.12%
24 ants	100.00%	93.72%	56.78%
32 ants	100.00%	100.00%	56.83%
40 ants	100.00%	100.00%	67.37%
48 ants	100.00%	100.00%	78.36%
56 ants	100.00%	100.00%	89.34%
64 ants	100.00%	100.00%	89.45%
72 ants	100.00%	100.00%	89.47%

**Table 4:** The average minimum generation.

	The minimum generation
RND	>10000
GA	54.23
SACO	>10000
ILPACO	161.34
IPVACO	135.26
IGPACO	47.89
ACIACO	24.12

**Table 5:** Coverage of TCAS by seven approaches.

	Statement coverage	Branch coverage	MC/DC
RND	69.37%	48.29%	12.12%
GA	87.82%	70.57%	15.47%
SACO	71.17%	49.77%	11.81%
ILPACO	85.89%	64.28%	17.64%
IPVACO	92.54%	72.68%	17.47%
IGPACO	89.28%	69.28%	17.68%
ACIACO	92.90%	75.11%	33.64%

**Table 6:** The coverage of different ant numbers on ACIACO.

ACIACO	Statement coverage	Branch coverage	MC/DC
8 ants	82.37%	64.30%	17.17%
16 ants	92.29%	75.88%	33.22%
24 ants	92.78%	78.68%	35.53%
32 ants	94.38%	80.23%	35.55%
40 ants	95.84%	84.29%	38.45%
48 ants	95.11%	82.30%	37.25%
56 ants	97.89%	87.77%	45.25%
64 ants	100.00%	92.87%	57.67%
72 ants	100.00%	96.45%	70.89%
80 ants	100.00%	100.00%	76.12%
88 ants	100.00%	100.00%	76.13%

**Table 7:** The minimum generation.

	The minimum generation
RND	>10000
GA	98.66
SACO	>10000
ILPACO	238.34
IPVACO	174.28
IGPACO	96.39
ACIACO	28.24
